# Toxicity of Macroalgae Extracts to Larvae of the Northern House Mosquito

**DOI:** 10.3390/life14121527

**Published:** 2024-11-21

**Authors:** Ahmed A. Rashed, Yasmin M. Heikal, Robert D. Finn, Mohamed H. Bayoumy, Amged El-Harairy, Dina A. Refaay

**Affiliations:** 1 Economic Entomology Department, Faculty of Agriculture, Mansoura University, Mansoura 35516, Egypt; 2 Botany Department, Faculty of Science, Mansoura University, Mansoura 35516, Egypt; 3 Department of Biochemistry & Medical Genetics, St. George’s International School of Medicine, Northumbria University, Newcastle upon Tyne NE1 8ST, UK; 4 Department of Crop and Animal Sciences, Albrecht Daniel Thaer-Institute of Agricultural and Horticultural Sciences, Faculty of Life Sciences, Humboldt-Universität zu Berlin, Albrecht-Thaer-Weg 5, 14195 Berlin, Germany; 5 Unit of Entomology, Plant Protection Department, Desert Research Center, 1 Mathaf El- Matariya St., El-Matariya, Cairo 11753, Egypt

**Keywords:** biocontrol, larvicidal activity, *Culex pipiens*, Ulva lactuca, *Turbinaria ornata*, genotoxicity, alkaline comet assay

## Abstract

The continuous use of synthetic insecticides to suppress mosquito larvae has detrimental impacts on the environment and human health. Finding novel and target-specific bio-insecticides has become crucial. Here, the larvicidal and genotoxic activities of different extracts from *Ulva lactuca* and *Turbinaria ornata* toward *Culex pipiens* larvae were investigated. The macroalgae thalli were subjected to various solvent extractions followed by phytochemical quantification, larvicidal testing on *C. pipiens* larvae, genotoxic evaluation through comet assays, and compound characterization by gas chromatography–mass spectrometry. The methylene chloride extract from *U. lactuca* displayed the highest toxicity with LC_50_ = 30.99 ppm, followed by the acetone extract from *T. ornata*, with LC_50_ = 52.09 ppm after 72 h. *U. lactuca* exhibited the maximum contents of total alkaloids, total flavonoids, total terpenoids, total phenols, and total tannins with the methanol extract, while the acetone extract from *T. ornata* exhibited the maximum contents of total alkaloids, total flavonoids, total terpenoids, and total phenols. The methylene chloride extract of *U. lactuca* and the acetone extract of *T. ornata* caused significant DNA damage in larva body cells. Thus, the methylene chloride extract from *U. lactuca* and the acetone extract from *T. ornata* showed promising potential as environmentally friendly larvicides against *C. pipiens* larvae.

## 1. Introduction

Mosquitoes are among the most important groups of insect vectors causing human diseases worldwide, transmitting diseases such as encephalitis, dengue fever, yellow fever, chikungunya, malaria, and Zika virus [1,2,3,4]. In Egypt, the mosquito *Culex pipiens* is the main vector of West Nile virus, Rift Valley fever virus, and lymphatic filariasis [5]. Traditional chemical insecticides such as organophosphates, carbamates, and pyrethroids are extensively used to control mosquito-associated diseases but negatively impact human health and the ecosystem [6]. Personal protection using mosquito nets while sleeping is effective only against mosquitoes that bite during the night and ineffective against mosquitoes with pesticide resistance [7,8].

In recent times, research has switched to the search for natural insecticides that are ecologically friendly and biodegradable but also target-specific. In this regard, marine macroalgae produce a wide variety of novel bioactive metabolites that could be used as eco-friendly alternatives to traditional insecticides in integrated pest management (IPM) programs [9,10]. One of the advantages of using marine algal extracts is their potential to be produced at a large scale cost-effectively [11]. Macroalgae are a rich source of highly diverse phytochemicals such as phenolic compounds, chlorophylls, carotenoids, flavonoids, alkaloids, terpenes, and phytosterols, with antioxidant, antimicrobial, and insecticidal activities [12].

Numerous studies have reported the effective mosquitocidal properties of several seaweeds and their extracts [13,14]. For instance, extracts from red seaweed, *Laurencia dendroidea*, have been reported to exhibit insecticidal activity against *Aedes aegypti* larvae [15]. The extracts of *Ulva lactuca* (Chlorophyta) showed an insecticidal effect on mosquito larvae and prevented the birth of new mosquitoes [16,17]. Methanolic extracts from *Gracilaria canaliculata* (formerly *Gracilaria crassa*) (Rhodophyta) and *Hypnea valentia* (Rhodophyta) showed effective larvicidal activity against mosquitoes [18]. Research has already shown effective larvicidal ability in *Ulva lactuca* (formerly *Ulva fasciata*) (Chlorophyta) and *Grateloupia lithophila* (Rhodophyta) extracts against *Culex quinquefasciatus* [19]. In addition to preventing mosquito larvae from growing and feeding and affecting metabolic activities, macroalga extracts have also shown increased effectiveness in the environment by preventing the larvae from breeding and dispersing [20]. Finally, the development of macroalga-derived bio-insecticides has been reported as a safe and cost-effective alternative for mosquito management [21].

The comet assay, often called the single-cell gel electrophoresis assay (SCGE), is a sensitive technique for identifying DNA damage. At first, the process involved reactions that occurred in neutral conditions, thereby only allowing for the detection of double-strand breaks in DNA [22]. The extension of the assay to alkaline conditions resulted in the ability to detect single-strand DNA damage [23], following which several modifications significantly increased the assay’s applications further. The comet assay is widely recognized as an easy, fast, and economical technique for assessing DNA damage, and, regardless of whether cells are proliferating or not, different cell types can be used in the test without prior knowledge of their karyotype and genome structure [24]. Eukaryotic organisms and cell types have also been tested with this assay [25,26]. The comet assay has been applied to cells of insects, including *Shistocerca gregaria*, *Drosophila melanogaster*, *Curculio sikkimensis*, mosquito larvae, and *Ephestia kuehniella*, and allows investigations into DNA damage in the different life stages of the mosquito *Culex pipiens* [27,28,29,30,31,32].

Based on the above information, the present work aimed to screen the larvicidal activity of macroalgae, *Ulva lactuca*, and *Turbinaria ornata* (Phaeophyceae) against the mosquito larvae *Culex pipiens* as a biocontrol agent. Second, we assessed the molecular genotoxicity of the most promising algal extracts through the comet assay. Third, we carried out quantitative analysis of the phytochemical constituents of the macroalga extracts in different solvent extracts.

## 2. Materials and Methods

### 2.1. Collection and Extraction of Macroalga Samples

*Turbinaria ornata* (Turner) J. Agardh was obtained from the Red Sea near Dahab city (28°34′19.99″ N, 34°32′14.55″ E) in Autumn, 2022, while *Ulva lactuca* Linnaeus was collected from Abo Qir, Alexandria, Egypt (31°19′00″ N 30°04′00″ E), in Summer, 2022. Specimen identification was performed by Prof. Dr. Mostafa M. El-Sheekh (Botany Department, Faculty of Science, Tanta University), and voucher specimens (*Turbinaria ornata*—Herb No. MU013 and *Ulva Lactuca*—Herb No. MU014) were deposited at the herbarium of Botany Department, Faculty of Science, Mansoura University, Egypt.

Alga samples were washed with seawater to remove sand particles and any epiphytes, and then 500 g fresh biomass from each specimen was transported to the phycology laboratory at Mansoura University’s Faculty of Science, where it was cleaned with tap water, allowed to dry in the free air in the shade at approximately 27 °C for several days until reaching a constant weight, and milled before being stored for further extraction.

Extraction was carried out using three different organic solvents, methanol (99.8%), acetone (99.9%), and dichloromethane (99.8%), each employed independently. A weight of 20 g of the appropriate macroalga powder was soaked in 200 mL of solvent for 48 h at room temperature under shaking at 200 rpm. The extracts were then dried and concentrated with a rotary evaporator, weighed, and kept at −4 °C for future use.

### 2.2. Rearing of Culex pipiens Mosquito

*Culex pipiens* is the commonest mosquito species in Egypt. Larvae were collected from several natural ponds in the Mansoura district using a rounded net dipper (20 cm in diameter) and transferred to the mosquito rearing lab at the Economic Entomology Department, Faculty of Agriculture, Mansoura University, under the lab conditions of a temperature of 27 ± 2 °C (using air conditioning), a relative humidity of 70 ± 10% (paper towels saturated with water only were placed on top of the cages once a day), and a 12/12 light/dark cycle. Larvae of *Culex pipiens* were morphologically identified using previously described keys [33,34]. Larvae were transferred to plastic cups and provided daily with yeast as a diet until pupation, with an increase in the amount in accordance with the development of the growth of larvae. The water was changed twice a week, and plastic strainers of various sizes and meshes were employed to separate the various larval instars or to change the water. Using a plastic dropper, pupae were separated, placed into plastic containers with fine mesh netting at the top, and moved to cages (30 × 30 × 30) until adults emerged. Adults were fed a 10% sucrose solution, and females were given access twice a week to a meal of pigeon blood using a membrane feeding system. After each blood meal, females laid eggs directly on the water surface in oviposition pots that were 1/5 filled with tap water. Egg rafts were transferred to experimental 30 × 30 × 6 cm plastic trays filled with 1.0 L of distilled water [1,35]. According to [36], mosquitoes were reared in the insectary for 10 generations before any experiments. For toxicity testing, third-instar larvae of *C. pipiens* were used.

### 2.3. Larvicidal Activity and Mortality Bioassay Test

The larvicidal efficiencies of the methanol, acetone, and methylene chloride extracts from *U. lactuca* and *T. ornata* against early 3rd-instar larvae of *C. pipiens* were determined according to the standard WHO protocol [37]. Each extract was dissolved in DMSO to prepare a graded series of concentrations (50 to 250 ppm). Mortality was recorded 24, 48, and 72 h post-treatment.

For toxicological testing, batches of 25 *C. pipiens* early-third-instar larvae were transferred to small disposable plastic cups (diameter 10 cm, height 5.5 cm) containing 100 mL of chlorine-free water and the appropriate concentration of each algal extract. For every experiment, three replicates were carried out, and DMSO was used as a control for each run.

The percentages of larval mortality were calculated for each test treatment concentration, with the mortality data being corrected according to [38], as follows:

$$\mathrm{Mortality}\;\%=\frac{X-Y}{X}\times 100$$

where X is survival in the untreated control and Y is survival in the treated sample.

The lethal concentrations (LC_25_, LC_50_, and LC_90_) were calculated using Probit analysis [39] and the statistics program LDP-line.

### 2.4. Phytochemical Analysis of Ulva lactuca and Turbinaria ornata Extracts

Total alkaloid content was determined using gallic acid as a standard according to the Paterson method [40], total phenolic content was determined using the Folin–Ciocalteu reagent method [41], and total flavonoid content was determined by following the colorimetric method [42], using quercetin as a standard, while the total terpenoid content in each algal extract was determined using the basic method of [43] with a slight modification established by [44], and linalool was used as a standard. Total tannin content was quantified using the methodology of [45], and tannic acid was used as a standard, while, finally, total saponin content was determined according to [46]. For each experiment, three replicates were carried out.

### 2.5. Chemical Characterization of Algae Extracts

A GC-TSQ mass spectrometer (Thermo Scientific, Austin, TX, USA) with a direct capillary column TG–5MS (30 m × 0.25 mm × 0.25 µm film thickness) was used for the gas chromatography–mass spectrometry (GC-MS) analysis of the extracts. The column oven temperature was initially maintained at 60 °C, then increased by 5 °C to 250 °C (maintained for 2 min.), and then finally increased to 300 °C by 30 °C/min. The injector temperature was maintained at 270 °C. Helium was used as a carrier gas at a constant flow rate of 1 mL/min. The solvent delay was 4 min, and diluted samples of 1 µL were injected automatically using Autosampler AS3000 coupled with GC in the split mode. EI mass spectra were collected at 70 eV ionization voltages over the range of *m/z* 50–650 in full scan mode. The ion source and transfer line were set at 200 °C and 280 °C, respectively. The components were identified via the comparison of their mass spectra with those of the WILEY 09 and NIST14 mass spectral databases.

### 2.6. Single-Cell Gel Electrophoresis Assay: Comet Assay for Rapid Genotoxicity Assessment

#### 2.6.1. Hemocyte Collection

The entire bodies of five mosquitos from the early-third-instar larvae of *C. pipiens* were washed with distilled water first, then sterilized with 5% bleach and dried. The cuticle was removed using two fine forceps. Hemolymph and hemocytes were collected in microcentrifuge tubes. Then, 20 µL of pooled hemolymph was centrifuged at 1000 rpm at 4 °C for 10 min. The supernatant was discarded, and the pellet was resuspended in 200 µL cold phosphate-buffered saline (PBS) for each sample. Three replicates were created, each with a pool of five larvae. According to [23], DNA damage was measured in *C. pipiens* whole body cells to assess the genotoxic effects of two significant algal extracts, the methylene chloride extract of *U. lactuca* and the acetone extract of *T. ornata*, compared to negative and positive controls (DMSO and commercial insecticide, Malathion 5), respectively.

#### 2.6.2. Comet Assay

Isolated hemocytes were suspended immediately in 50 µL of ice-cold Ringer solution. Then, 10 µL of isolated cells was mixed with 90 µL of 1% low-melting-point agarose (LMPA) and placed on microscope slides pre-coated with 1.5% NMA (normal melting agar). The slides were immediately placed on ice following the attachment of a cover slip. After the agarose solidified, the solids were immersed for 24 h at 4 °C in a lysis solution (0.25 M NaOH, 2.5 M NaCl, 10 mM Tris, 1% Triton X-100, 100 mM EDTA, and 10% DMSO, pH = 10.0) before being immersed in electrophoresis buffer (300 mM NaOH and 1 mM EDTA, pH = 13) for 20 min in a horizontal gel electrophoresis tank. Electrophoresis was carried out for 20 min at 24 V and 270 mA at 4 °C. The slides were fixed with methanol and allowed to dry overnight at room temperature after neutralization with 0.4 M Tris-HCl (pH = 7.4) and before staining with (2 µg/mL) ethidium bromide (EtBr). Comets were viewed at 400× magnification using an Axio fluorescence microscope (Carl Zeiss, Oberkochen, Germany) with an excitation filter of 524 nm and a barrier filter of 605 nm. Then, 50 to 100 randomly chosen cells were examined using the Kinetic Imaging, Ltd. (Liverpool, UK) Komet 5 image analysis program, coupled to a CCD camera.

### 2.7. Statistical Analysis

Data were analyzed by one-way analysis of variance (ANOVA) and Duncan’s multiple-range method. Values were expressed as means ± standard deviation. Differences were considered significant at *p* < 0.05. All analyses were performed in triplicate. Lethal concentrations (LC_25_, LC_50_, and LC_90_) with 95% fiducial limits for the upper and lower confidence limits, Chi-square, slope, standard error, and confidence intervals were calculated by probit analysis [39] using the statistics package LDP-line. In the comet assay, the DNA damage in *C. pipiens* larva cells was examined using three distinct types of DNA damage measurements: the length of the DNA comet tail (TL), the percentage of fragmented DNA in the tail (% TD), and the tail moment (TM) after electrophoresis. Histograms and one-sample *t*-test were estimated and plotted as three replicates using GraphPad Prism 9 (GraphPad Software, Inc., San Diego, CA, USA). Normality was assessed using Shapiro–Wilk normality testing at *p* < 0.05. A cell plot of all analyzed parameters in response to the *U. lactuca* and *T. ornata* extracts was prepared using JMP^®^, Version 17.2.0 (SAS Institute Inc., Cary, NC, USA, 2022–2023).

## 3. Results

### 3.1. Larvicidal Activity

The data presented in Table 1, Table 2 and Table 3 demonstrate the larvicidal activities of the acetone, methanol, and methylene chloride extracts of *U. lactuca* and *T. ornata* against 3rd-instar larvae of *Culex pipiens* at 24, 48, and 72 h post-treatment. The data indicate that the toxicity of the different extracts varies according to the macroalgal species, with the mortality of mosquito larvae being initiated from day 1 of exposure and increasing up to day 3, depending on the extract. For the acetone extracts, the highest toxicity was recorded for *T. ornata*, with LC_50_ values of 126.24, 79.14, and 52.09 ppm after 24, 48, and 72 h, respectively (Table 1). For the methylene chloride and methanol extracts (Table 2 and Table 3), *U. lactuca* was the most toxic genus, with LC_50_ values of 62.24, 37.19, and 30.99 ppm after 24, 48 and 72 h, respectively, in the case of the methylene chloride extract, and LC_50_ values of 257.37, 134.06, and 72.57 ppm after 24, 48, and 72 h, respectively, in the case of the methanol extract.

Table 4 reports the mortality percentage for the macroalga *U. lactuca*, revealing the highest mortality after 24, 48, and 72 h for the methylene chloride extract (90, 96.67 and 100% at 250 ppm, respectively); 100% mortality was also observed for this alga extract after 72 h at concentrations of 150 ppm and 200 ppm. The acetone extract for *T. ornata* resulted in 100% mortality at 250 ppm. The lowest mortality percentage was observed for the methanol and methylene chloride extracts of *T. ornata*, at 53.33% and 63.33%, respectively.

### 3.2. Evaluation of DNA Damage

**Figure 1:**
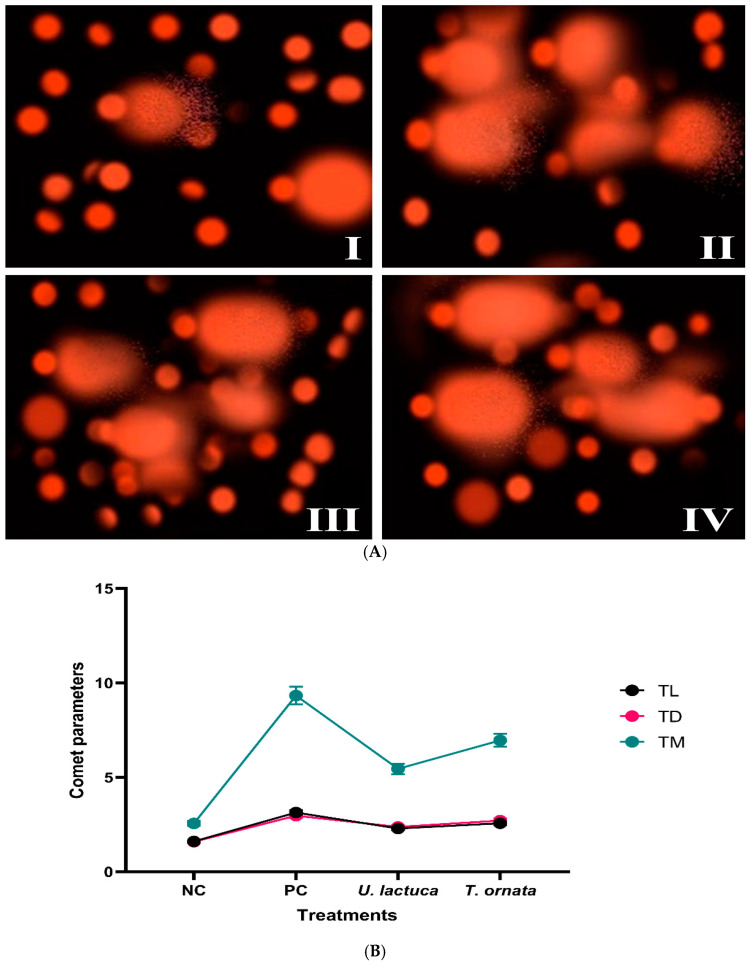
Comet assay for genotoxicity. (**A**) Comparison of comet profiles in early-third-instar larvae of *Culex pipiens* under different treatments. (**B**) Comet assay parameters (tail length (TL), tail DNA (TD), and tail moment (TM) of early-third-instar larvae of *C. pipiens* under different treatments. (**AI**) NC, negative control (DMSO); (**AII**) PC, positive control (Malathion 5); (**AIII**) *Ulva lactuca* (methylene chloride extract); (**AIV**) *Turbinaria ornata* (acetone extract).

DNA damage was assessed in *C. pipiens* cells following treatment with each of the two best performing algal extracts, i.e., the methylene chloride extract of *U. lactuca* and the acetone extract of *T. ornata*. DNA damage was quantified using the comet test and the following parameters of tail length (TL), tail DNA% (TD), and tail moment (TM). Differences in the degree of DNA damage were observed and recorded between treated and control *C. pipiens* larvae, regardless of which extract was used (Figure 1). In the negative control (DMSO), body cells appeared as rounded nuclei (Figure 1AI). Nuclei with a visible tail-like extension were observed in the body cells of mosquitoes treated with the positive control (Malathion 5), indicating that the insect’s body cells had been damaged and DNA strand fragmentation had occurred (Figure 1AII). Insecticide treatment was observed to generate the most significant increase in the levels of DNA damage in *C. pipiens* body cells with an increase in TL of 3.14 µm compared to the negative control (1.61 µm), tail DNA% values of 2.97% compared to the negative control (1.59%), and a considerable rise in the values of TM, 9.33 compared to the control values (2.56), as shown in Figure 1B. Figure 1AIII,IV,B show DNA damage in *C. pipiens* body cells treated with the methylene chloride extract of *U. lactuca* and acetone extract of *T. ornata*. For *U. lactuca* and *T. ornata*, the TL values were 2.29 and 2.57 µm, TD% was 2.38 and 2.71%, and the TM values were 5.45 and 6.96, respectively.

### 3.3. Extraction Yield

**Figure 2:**
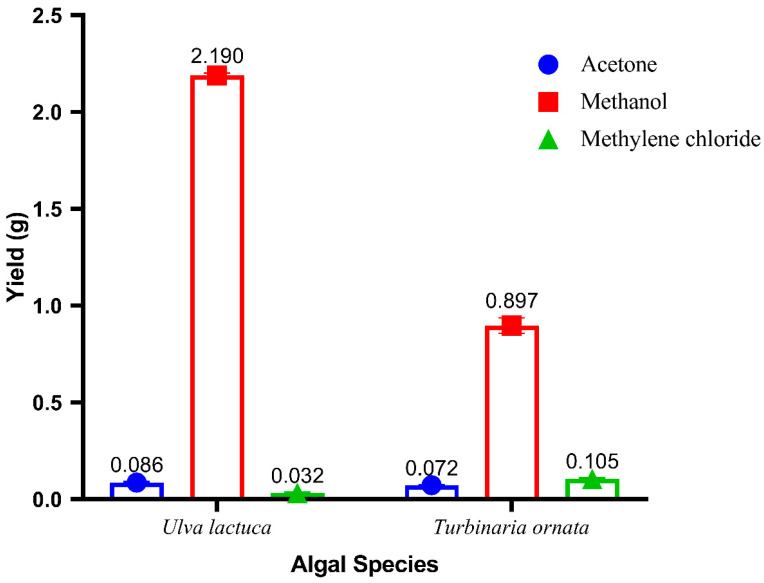
The average yield (g) of each solvent extract (acetone, methanol, and methylene chloride) from *Ulva lactuca* and *Turbinaria ornata*.

Figure 2 represents the mean values of the yield (g) of each solvent extract from *U. lactuca* and *T. ornata*. The methanolic extract of *U. lactuca* had the highest yield (2.19 ± 0.012 g) and the methylene chloride extract had the lowest yield (0.032 ± 0.001 g). Similarly, *T. ornata* recorded the maximum yield in the case of the methanol extract (0.897 ± 0.040 g) and the lowest yield (0.072 ± 0.001 g) in the case of the acetone extract.

### 3.4. Phytochemical Analysis

**Figure 3:**
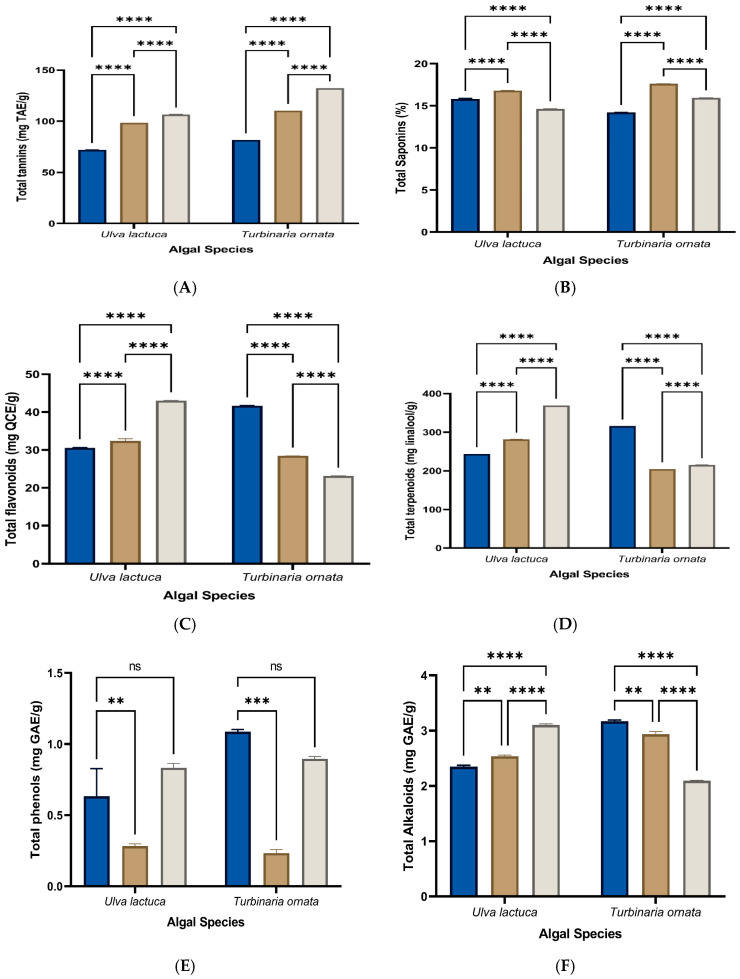
Changes in (**A**) total tannins, (**B**) saponin contents, (**C**) total flavonoids, (**D**) total terpenoid contents, (**E**) total phenols, and (**F**) alkaloids in different solvent extracts from both *U. lactuca* and *T. ornata*. Data represent the mean ± SD, *n* = 3. Two-way ANOVA according to Tukey’s multiple-comparisons test. ns = non-significant, **, ***, and **** refer to two means significantly different at the 0.01, 0.001, and ≤0.0001 levels.

Figure 3A–F displays the yields (mg/g extract) of the total amount of tannins, saponins, flavonoids, terpenoids, phenolics, and alkaloids for the different crude extracts isolated from *U. lactuca* and *T. ornata*. For the green macrophyte *U. lactuca*, the highest tannin content of 106.63 ± 0.208 mg tannic acid equivalent (TAE)/g was obtained in the methylene chloride extract and the lowest (72.09 ± 0.060 mg TAE/g) was obtained in the acetone extract. Similarly, for *T. ornata*, the highest tannin content (132.51 ± 0.026 mg TAE/g) was obtained in the methylene chloride extract, and the lowest (81.69 ± 0.03 mg TAE/g) was obtained in the acetone extract (Figure 3A). *U. lactuca* maintained the highest saponin content (16.81 ± 0.025%) in the methanol extract and the lowest (14.64 ± 0.026%) in the methylene chloride extract. *T. ornata* maintained the highest saponin content (17.62 ± 0.030%) in the methanol extract and the lowest (14.21 ± 0.02%) in the acetone extract (Figure 3B).

The highest total flavonoid content of 43.02 ± 0.02 mg quercetin equivalent (QCE)/g was found in the methylene chloride extract of *U. lactuca*, while the lowest (30.58 ± 0.036 mg QCE/g) was obtained for the acetone extract. For *T. ornata*, the highest total flavonoid content (41.68 ± 0.04 mg QCE/g) was found in the acetone extract and the lowest (23.44 ± 0.03 mg QCE/g) was found in the methylene chloride extract (Figure 3C). Similarly, *U. lactuca* showed the highest terpenoid content (369.2 ± 0.02 mg linalool/g) in the methylene chloride extract and the lowest (243.85 ± 0.015 mg linalool/g) in the acetone extract. Meanwhile, in *T. ornata*, the highest total terpenoid content (316.063 ± 0.025 mg linalool/g) was found in the methylene chloride extract and the lowest (204.62 ± 0.02 mg linalool/g) was recorded for the methanol extract (Figure 3D).

*U. lactuca* displayed the highest total phenolic content (0.833 ± 0.03 mg gallic acid equivalent (GAE)/g) for the methylene chloride extract and the lowest (0.283 ±0.015 mg GAE/g) for the methanol extract. In *T. ornata*, the highest total phenolic content (1.086 ± 0.015 mg GAE/g) was found in the acetone extract and the lowest (0.233 ± 0.025 mg GAE/g) was found in the methanol extract (Figure 3E). *U. lactuca* showed the highest total alkaloid content (3.1 ± 0.02 mg GAE/g) in the methylene chloride extract and the lowest (2.346 ± 0.015 mg GAE/g) in the case of the acetone extract. The highest total alkaloid content (3.16 ± 0.025 mg GAE/g) was found in the acetone extract for *T. ornata* and the lowest (2.09 ± 0.005 mg GAE/g) was found in the methylene chloride extract (Figure 3F).

### 3.5. GC-MS Analysis

For the *U. lactuca* methylene chloride extract, the GC chromatogram (Figure S1) exhibited 23 peaks corresponding to 23 compounds, of which only 16 compounds could be characterized and identified (Table 5. The four major peaks were identified as n-hexadecanoic acid (50.14%), phytol (9.039%), isochiapin B (5.34%), and 2-Pentadecanone, 6,10,14-trimethyl- (4.27%). The GC chromatogram (Figure S2) of the *T. ornata* acetone extract contained 35 peaks corresponding to 35 phytochemical compounds, of which only 14 compounds could be characterized and identified. Table 6 shows that the five major peaks in the extract were identified as hexadecanoic acid, methyl ester (37.5%), n-hexadecanoic acid (22.57%), l-(+)-Ascorbic acid 2,6-dihexadecanoate (11.03%), hexadecyl nonyl ether (6.53%), and indole-2-one,2,3-dihydro-N-hydroxy-4-methoxy-3,3-dimethyl- (5.56%).

### 3.6. Cell Plot of U. lactuca and T. ornata Extract Composition and Activities

**Figure 4:**
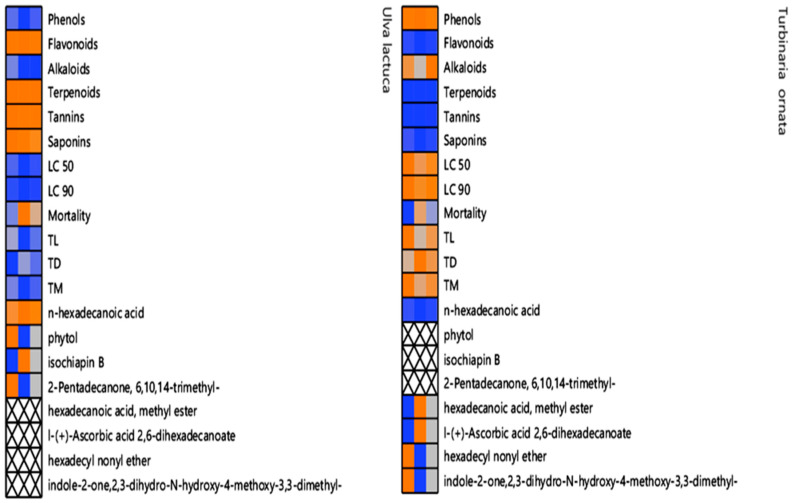
Cell plot of 20 phytochemical traits and some selected metabolites for GC/MS analysis of *U. lactuca* (methylene chloride extract) and *T. ornata* (acetone extract) and their larvicidal activity (LC_50_ and LC_90_; larvicidal potency and mortality percentage), comet parameters (TL, tail length; TD, tail DNA; and TM, tail moment) in early-third-instar larvae of *C. pipiens.* The orange color shows the highest value, while the blue color shows the lowest one.

As shown in Figure 4, the cell plot of 20 studied (phytochemical) parameters and some selected metabolites for the GC-MS analysis of *U. lactuca* (methylene chloride extract) and *T. ornata* (acetone extract) and their larvicidal activity (LC_50_ and LC_90_; larvicidal potency and mortality percentage), comet parameters (TL, tail length; TD, tail DNA; and TM, tail moment) were assessed in early-third-instar larvae of *C. pipiens*; the orange color shows the highest value, while the blue color shows the lowest. For phytochemical analysis, the acetone extract of *T. ornata* showed the maximum values for phenols, alkaloids, LC_50_, LC_90_, TL, TD, and TM, while the methylene chloride extract of *U. lactuca* had the highest values for flavonoids, terpenoids, tannins, and saponins. Based on the GC-MS profiling data, some important metabolites differed in their levels between the algal extracts with hexadecanoic acid, methyl ester, l-(+)-Ascorbic acid 2,6-dihexadecanoate, hexadecyl nonyl ether, and indole-2-one,2,3-dihydro-N-hydroxy-4-methoxy-3,3-dimethyl- being found in the *T. ornata* acetone extract only, whereas n-Hexadecanoic acid, phytol, isochiapin B, and 2-Pentadecanone, 6,10,14-trimethyl- were found only in the *U. lactuca* methylene chloride extract. In terms of larvicidal potency (LC_50_ and LC_90_), the *T. ornata* acetone extract outperformed the *U. lactuca* methylene chloride extract; however, the latter had the highest mortality rate, and the *T. ornata* acetone extract had the greatest genotoxicity scores for the three comet criteria (TL, TD, and OM).

## 4. Discussion

Using bio-insecticides as an alternative to chemical insecticides is the focus of many researchers who are seeking eco-friendly control tools to overcome the adverse health effects on humans and the ecosystems [47]. Macroalga-derived bio-insecticides are among the range of bioactives that have been reported as safe and cost-effective alternatives, with many researchers showing that macroalgae have toxic effects on different insects, especially mosquitoes [13,20].

Accordingly, the present study was designed to investigate the potential of different solvent extracts from the macroalgae *Ulva lactuca* and *Turbinaria ornata* as biocontrol tools against the larvae of the mosquito *Culex pipiens* L. The experimental data presented here revealed that the larvicidal activities of the test macroalgae against the third larval instar of *C. pipiens* varied according to the solvent used for extraction and the macroalgae species, in agreement with the findings of [48] and those of [49], who showed that green algal extracts possessed insecticidal activities against third-instar larvae of *C. pipiens.*

Herein, the methylene chloride extract from *U. lactca* was determined to be the most toxic, with an LC_50_ = 30.99 mg L^−1^, followed by the acetone extract from *T. ornata*, with a LC_50_ = 52.09 mg L^−1^ for an exposure time of 72 h. These results are in agreement with the findings of [50], who similarly showed acetone extracts to be the most effective against the fourth-instar larvae of *C. pipiens*, as well as the study of [16], in which the acetone extract of *U. lactuca* was the most effective extract against *C. pipiens* larvae, and, additionally, these results are in partial agreement with several other studies [17,51]

According to the classification of [52], plant extracts are considered effective larvicides with an LC_50_ < 100 mg L^−1^, while extracts with an LC_50_ > 200 mg L^−1^ are considered ineffective larvicides. Thus, based on this classification, the *T. ornata* acetone extract and the *U. lactuca* methylene chloride extract are considered effective for larvicidal biocontrol. This classification is further supported by the finding that 100% larval mortality was observed for the *U. lactuca* methylene chloride extract at concentrations of 150, 200, and 250 ppm and for the *T. ornata* acetone extract at a concentration of 250 ppm against the third larval instar of *C. pipiens*. The data presented here are also in agreement with those obtained by [15], who demonstrated that at a concentration of 50 ppm, the dichloromethane and the methanol extracts from the red seaweed *Laurencia dendroidea* caused 100% mortality against the fourth-instar larvae of *Aedes aegyptii.* Similar results were reported by [53] for the larvicidal activity against the third-instar larvae of *Culex pipens*. In addition, the larvicidal mortality rate increased with the exposure time. Acetone extracts of several seaweeds, including *U. lactuca*, were active against the fourth-instar larvae of *A. aegypti* [54]. Finally, [55] reported that the chloroform extract of *Ulva lactuca* (formerly *Ulva fasciata*) displayed 100% mortality at a concentration of 500 mg/L against larval instars of *Culex quinquefasciatus*.

The larva mortality rates exhibited by the different extracts may be attributed to the biomolecules identified in each extract that have been reported previously for their insecticidal, pesticide, nematicide, and antioxidant activity. Based on multivariate analyses, mortality rates for the *T. ornata* acetone extract showed a strong positive correlation with levels of hexadecanoic acid, methyl ester, and l-(+)-Ascorbic acid 2,6-dihexadecanoate in GC-MS metabolites. For the methylene chloride extract of *U. lactuca*, n-hexadecanoic acid and isochiapin B. showed strong positive correlations with mortality percentages in third-instar larvae of *C. pipens*. These findings are in agreement with the findings of several researchers who determined similar correlations for 2,4,4,6,6,8,8-Heptamethyl-2-nonene, l-(+)-Ascorbic acid 2,6-dihexadecanoate, hexadecanoic acid, methyl ester, and n-hexadecanoic acid [56,57,58,59,60]. Fatty acids and their methylated forms have also been shown to have insecticidal activities against *Culex quinquefasciatus*, as reported by [61]. The identified compounds from the methanolic extract of *U. lactuca* revealed the presence of 8-heptadecene, 9-octadecenoic acid, n-hexadecanoic acid, and phytol, which has been reported in the literature to have insecticide, pesticide and anti-feedant activities [62,63,64].

Additionally, the activities of the different extracts could be due to the specific combination of biochemical constituents, which includes phenolic, terpenoids, flavonoids, saponins, tannins, and alkaloids that exist in the test macroalga biomass. These compounds may jointly or independently contribute to the larvicidal activities against *C. pipiens* larvae. In support of this, it has been reported by [65] that mixes of saponins serve as natural larvicidal compounds, by [66] that mixes of tannins have larvicidal and repellant qualities that affect the growth, development, and fecundity of several phytophagous insects, and by [67] that mixes of phenols, alkaloids, terpenes, tannins, flavonoids saponins, and anthraquinones isolated from macroalgae are associated with insecticidal and repellent activity. The phenolic compound from *Limnospira platensis* (formerly *Arthrospira platensis*) (Cyanobacteria) was documented by [68], and [69] showed the insecticidal activity. Finally, algal extracts have been shown to exhibit different insecticidal effects, depending on the insect species and the developmental stages of the target insect, as documented by [16], which showed how ethanolic and chloroform extracts from *U. lactuca* acted as larvicides in the case of the cotton leafworm, *Spodoptera littoralis*; methanolic and ethanolic extracts caused the highest pupation inhibition, while etheric and methanolic extracts strongly inhibited larval growth and adult emergence. These reports therefore support the suggestion that larvicides represent effective strategies for controlling the population of mosquitoes, since controlling the larvae that live in bounded aquatic areas is easier than targeting the free-flying adults [70].

The comet assay has been successfully applied to cells of insects, including *Shistocerca gregaria*, *Drosophila melanogaster*, *Curculio sikkimensis*, mosquito larvae, and *Ephestia kuehniella* [27,28,29,30,31]. The comet assay is regarded as one of the most significant tests for determining genotoxicity in fish and aquatic insects following exposure to water contaminants, either in the environment or under experimental laboratory treatments [30,71], which, together with the findings of [32], who investigated DNA damage in the third- and fourth-larval instars, pupae, and male and female adults of mosquito *Culex pipiens* collected from two contaminated water streams (Nikla and Elmansoreyh), confirms that the comet assay used here is a suitable and sensitive tool for the environmental monitoring of the on-target effects of the macroalga extracts studied in this report.

## 5. Conclusions

It can be concluded that the methylene chloride extract from *U. lactuca* and the acetone extract from *T. ornata* displayed effective larvicidal activity against the third-instar larvae of *C. pipiens* with low LC_50_ values. The methylene chloride extract from *U. lactuca* was found to be the most toxic, resulting in 100% mortality in *C. pipiens* at concentrations of 250, 200, and 150 ppm after 72 h. DNA damage in *C. pipiens* body cells treated with both the methylene chloride extract of *U. lactuca* and the acetone extract of *T. ornata* was observed to be high. Mosquitoes fed with the acetone extract of *T. ornata* had the greatest values for three comet assay parameters, TL, TD, and TM, compared to the controls. The GC-MS analyses of the *U. lactuca* methylene chloride extract and the *T. ornata* acetone extract revealed the presence of phytochemicals known to possess insecticidal activity. Based on the data presented here, these extracts have the potential to be used as environmentally friendly larvicides against *C. pipiens.* However, it is clear that further research is needed to develop a novel formulation, a “nanoformulation”, suitable for application in a field scenario to decipher the underlying toxicology mechanisms leading to the observed activity.

## Figures and Tables

**Table 1:** Larvicidal activity of the acetone extracts from *U. lactuca* and *T. ornata* against early-third-instar larvae of *Culex pipiens*.

Macroalga Species	Post-Treatment	LC_25_ (* F.l. at 95%)	LC_50_ (* F.l. at 95%)	LC_90_ (* F.l. at 95%)	* Slope ± SE	*p*	* X^2^	Toxicity Index	Relative Potency
*U. lactuca*	24 h	134.13	223.81	592.09	3.033 ± 0.348	0.388	3.023	23.27	1
		(119.14–147.99)	(200.95–258.03)	(459.45–876.27)					
	48 h	91.84	154.470	414.84	2.987 ± 0.291	0.350	3.281	33.71	1.45
		(79.13–102.94)	(140.74–170.32)	(343.49–542.50)					
	72 h	58.50	104.86	317.85	2.661 ± 0.261	0.0014	15.625	49.67	2.13
		(46.80–73.13)	(83.89–131.08)	(254.28–397.31)					
*T. ornata*	24 h	58.35	126.24	547.05	2.013 ± 0.248	0.661	1.595	41.26	1.77
		(43.11–71.29)	(109.82–144.16)	(405.52–879.39)					
	48 h	36.624	79.14	342.1	2.016 ± 0.247	0.059	7.458	65.82	2.83
		(24.27–47.69)	(64.44–92.13)	(270.95–489.39)					
	72 h	26.32	52.09	190.53	2.276 ± 0.275	0.0059	12.472	100	4.29
		(21.06–32.89)	(41.67–65.11)	(152.43–238.16)					

* (F.l.) fiducial limit. * (X^2^) Chi-square value. * Slope of the concentration–inhibition regression line ± standard error.

**Table 2:** Larvicidal activity of the methanol extracts from *U. lactuca* and *T. ornata* against early-third-instar larvae of *C. pipiens*.

Macroalga Species	Post-Treatment	LC_25_ (* F.l. at 95%)	LC_50_ (* F.l. at 95%)	LC_90_ (* F.l. at 95%)	* Slope ± SE	*p*	* X^2^	Toxicity Index	Relative Potency
*U. lactuca*	24 h	113.95	257.37	1210.19	1.906 ± 0.279	0.0085	11.692	28.2	6.19
		(91.16–142.44)	(205.89–321.71)	(968.16–1512.75)					
	48 h	59.66	134.06	624.29	1.918 ± 0.248	0.0086	11.6737	54.14	11.89
		(47.73–74.58)	(107.25–167.58)	(499.43–780.37)					
	72 h	35.45	72.58	283.13	2.168 ± 0.252	0.002	14.768	100	21.96
		(28.36–44.32)	(58.06–90.723)	(226.50–353.91)					
*T. ornata*	24 h	469.03	1594.04	16,292.93	1.269 ± 0.379	0.726	1.313	4.55	1
		(375.23–586.29)	(1275.235–1992.555)	(13,034.3424–20,366.16)					
	48 h	146.93	603.34	8833.15	1.099 ± 0.270	0.2576	4.0358	12.03	2.64
		(110.73–198.88)	(482.668–754.1687)	(7066.5224–11,041.441)					
	72 h	42.12	263.43	8577.65	0.847 ± 0.237	0.4679	2.5409	27.55	6.05
		(33.69–52.65)	(210.741–329.2837)	(6862.1176–10,722.0587)					

* (F.l.) fiducial limit. * (X2) Chi-square value. * Slope of the concentration–regression line ± standard error.

**Table 3:** Larvicidal activity of the methylene chloride extracts from *U. lactuca* and *T. ornata* against early-third instar larvae of *C. pipiens*.

Macroalga Species	Post-Treatment	LC_25_ (* F.l. at 95%)	LC_50_ (* F.l. at 95%)	LC_90_ (* F.l. at 95%)	* Slope ± SE	*p*	* X^2^	Toxicity Index	Relative Potency
*U. lactuca*	24 h	26.98	62.24	304.65	1.858 ± 0.248	0.407	2.903	49.79	8.89
		(15.49–37.76)	(46.68–75.42)	(240.34–443.27)					
	48 h	14.6	37.19	219.01	1.664 ± 0.272	0.311	3.574	83.33	14.89
		(5.54–24.43)	(21.39–50.59)	(172.57–326.26)					
	72 h	18.264	30.99	84.65	2.937 ± 0.434	0.009	11.551	100	17.87
		(14.61–22.83)	(24.79–38.74)	(67.72–105.81)					
*T. ornata*	24 h	250.73	553.88	2497.09	1.959 ± 0.385	0.297	3.693	5.59	1
		(206.87–352.06)	(383.31–1221.28)	(1997.6–3121.3)					
	48 h	124.84	299.67	1582.07	1.774 ± 0.282	0.742	1.246	10.34	1.85
		(102.87–146.11)	(240.81–433.97)	(1265.6–1977.5)					
	72 h	51.66	167.31	1560.73	1.322 ± 0.239	0.603	1.857	18.52	3.31
		(28.93–69.97)	(137.54–216.84)	(1248.58–1950.90)					

* (F.l.) fiducial limit. * (X^2^) Chi-square value. * Slope of the concentration–inhibition regression line ± standard error.

**Table 4:** Mortality percentage of *U. lactuca* and *T. ornata* extracts against early-third-instar larvae of *C. pipiens* at different concentrations.

Macroalga Species	% Mortality ± SD											
	Acetone				Methanol				Methylene Chloride			
	Conc. (ppm)	24 h	48 h	72 h	Conc. (ppm)	24 h	48 h	72 h	Conc. (ppm)	24 h	48 h	72 h
*U. lactuca*	250	53.33 ± 5.77	76.67 ± 5.77	93.33 ± 11.5	250	60 ± 10	80 ± 10	96.67 ± 5.77	250	90 ± 10	96.67 ± 5.77	100 ± 0
	200	46.67 ± 11.55	63.3 ± 15.28	73.33 ± 5.77	200	36.67 ± 15.27	60 ± 10	80 ± 10	200	83.3 ± 5.77	93.3 ± 5.77	100 ± 0
	150	33.33 ± 5.77	46.67 ± 5.77	63.33 ± 5.77	150	26.67 ± 5.77	46.67 ± 5.77	70 ± 10	150	73.3 ± 5.77	83.3 ± 5.77	100 ± 0
	100	10 ± 10	23.3 ± 5.77	36.67 ± 5.8	100	16.67 ± 5.77	33.33 ± 5.77	53.33 ± 5.77	100	60 ± 10	73.3 ± 11.55	86.67 ± 5.77
	50	3.3 ± 5.77	10 ± 10	26.67 ± 11.5	50	13.33 ± 5.77	26.67 ± 11.55	43.33 ± 15.3	50	46.67 ± 5.77	60 ± 10	76.67 ± 5.77
*T. ornata*	250	73.3 ± 5.77	86.67 ± 5.77	100 ± 0	250	16.67 ± 5.77	40 ± 10	53.33 ± 5.8	250	30 ± 10	46.67 ± 5.77	63.33 ± 5.77
	200	66.67 ± 5.77	80 ± 0	96.67 ± 5.77	200	13.33 ± 5.77	26.67 ± 5.77	46.67 ± 5.8	200	16.67 ± 5.77	36.67 ± 5.77	53.3 ± 5.77
	150	56.67 ± 5.77	73.3 ± 5.77	68.67 ± 5.77	150	6.67 ± 5.77	20 ± 10	36.67 ± 11.5	150	10 ± 0	30 ± 10	43.3 ± 5.77
	100	36.67 ± 5.77	46.67 ± 5.77	63.33 ± 11.5	100	6.67 ± 5.77	20 ± 10	33.33 ± 15.3	100	6.67 ± 5.77	16.67 ± 5.77	36.67 ± 5.77
	50	23.3 ± 5.77	40 ± 0	53.33 ± 5.77	50	3.3 ± 5.77	13.3 ± 5.77	30 ± 10	50	3.3 ± 5.7	10 ± 10	26.67 ± 5.77

**Table 5:** GC-MS analysis of the methylene chloride extract from *U. lactuca*.

No.	Peak No.	Compound	Chemical Group	Retention Time (min.)	Formula	Molecular wt.	Area %
1	1	8-Heptadecene	Alkene	26.8	C_17_H_34_	238	3.34
2	2	Tetradecanoic acid	Fatty acid	22.43	C_14_H_28_O_2_	228	1.14
3	3	2-Pentadecanone, 6,10,14-trimethyl-	Terpene	24.01	C_18_H_36_O	268	4.27
4	4	Phytol, acetate	Diterpene	24.16	C_22_H_42_O	338	3.88
5	6	3,7,11,15-Tetramethyl-2-hexadecen-1-ol	Terpenoid	24.64	C_20_H_40_O	296	2.75
6	7	Erucic acid	Fatty acid	25.46	C_22_H_42_O_2_	338	3.06
7	8	Hexadecanoic acid, methyl ester	Fatty acid methylester	25.62	C_17_H_34_O	270	2.55
8	9	n-Hexadecanoic acid	Fatty acid	26.66	C_16_H_32_O_2_	256	50.14
9	10	9-Octadecenoic acid	Fatty acid	27.1	C_18_H_34_O_2_	282	2.64
10	12	Phytol	Diterpene	29.17	C_20_H_40_O	296	9.039
11	13	*cis*-13-Eicosenoic acid	Fatty acid	29.54	C_20_H_38_O_2_	310	2.18
12	15	*Z*-(13,14-Epoxy)tetradec-11-en-1-ol	Alcohol	31.26	C_16_H_28_O_3_	268	2.1
13	16	Tricyclo[20.8.0.0(7,16)]triacontane,	Terpene	33.93	C_30_H_52_O_2_	444	1.21
		1(22),7(16)-diepoxy-					
4	17	Hexadecanoic acid, 2,3-dihydroxypropyl ester	Fatty acid methylester	35.96	C_19_H_38_O_4_	330	2.19
15	19	Oleic acid, eicosyl ester	Fatty acid methylester	41.39	C_38_H_74_O	562	3.48
16	21	Isochiapin B	Sesquiterpene lactone	44.5	C_19_H_22_O_6_	336	5.34

**Table 6:** GC-MS analysis of the acetone extract from *T. ornata*.

No.	Peak No.	Compound	Chemical Group	Retention Time (min.)	Formula	Molecular Weight	Area %
1	1	*E*,*E*,*Z*-1,3,12-Nonadecatriene-5,14-diol	Alcohol	26.09	C_19_H_34_O_2_	294	0.92
2	2	2,4,4,6,6,8,8-Heptamethyl-2-nonene	Ketone	26.34	C_16_H_32_	224	2.7
3	3	l-(+)-Ascorbic acid 2,6-dihexadecanoate	Oil-soluble vitamin C	26.53	C_38_H_68_O_8_	652	11.03
4	4	Isochiapin B	Sesquiterpene lactone	27.555	C_19_H_26_O_6_	336	1.6
5	5	Carbonic acid, prop-1-en-2-yl tridecyl ester	Fatty acid ester	27.757	C_17_H_32_O_3_	284	0.92
6	6	1H-Indole-2-carboxylic acid, 6-(4-ethoxyphenyl)-3-methyl-4-oxo-4,5,6,7-tetrahydro-, isobutyl ester	Indole alkaloid	28.088	C_23_H_29_NO_5_	399	2.035
7	7	Hexadecanoic acid, methyl ester	Fatty acid methylester	28.485	C_17_H_34_O	270	37.5
8	8	n-Hexadecanoic acid	Fatty acid	28.821	C_16_H_32_O_2_	256	22.57
9	9	Methyl stearate	Fatty acid methylester	30.238	C_19_H_38_O_2_	298	4.28
10	12	Hexadecyl nonyl ether	Ether	30.924	C_25_H_52_O	368	6.53
11	14	Hexahydropyridine,1-methyl-4-[4,5-dihydroxyphenyl]-	Alkaloid	32.975	C_12_H_17_NO_2_	207	1.39
12	15	2- methyltetracosane	Hydrocarbon	33.165	C_25_H_52_	352	1.21
13	18	Dodecahydropyrido [1,2-b]isoquinolin-6-one	Alkaloid	34.675	C_13_H_21_NO	207	2.35
14	25	Indole-2-one,2,3-dihydro-N-hydroxy-4-methoxy-3,3-dimethyl-	Alkaloid	37.207	C_11_H_13_NO_3_	207	5.56

## Data Availability

All data generated or analyzed during this study are included in this published article.

## Supplementary Materials

The following supporting information can be downloaded at: https://www.mdpi.com/article/10.3390/life14121527/s1, Figure S1. GC-MS chromatogram: (A) *U. lactuca* (methylene chloride extract) structure, formula, molecular weight, CAS number, and the relative abundance of the four most abundant metabolites; (B) n-Hexadecanoic acid; (C) 2-Pentadecanone, 6,10,14-trimethyl-; (D) Phytol; (E) Isochiapin B. Figure S2. (A) GC-MS chromatogram of acetone extract of *T. ornata* and the formula, molecular weight, CAS number, and the relative abundance of the most abundant metabolites: (B) Hexadecanoic acid, methyl ester, (C) n-Hexadecanoic acid, (D) L-(+)-Ascorbic acid 2,6-dihexadecanoate, (E) Indole-2-one,2,3-dihydro-N-hydroxy-4-methoxy-3,3-dimethyl-, and (F) Hexadecyl nonyl ether.
