# Supplementary material for: Toxicity of Macroalgae Extracts to Larvae of the Northern House Mosquito
Source: Life (Basel). 2024 Nov 21;14(12):1527. doi: 10.3390/life14121527 (PMC11677908; doi:10.3390/life14121527)
Supplement: Supplementary file 1 [file life-14-01527-s001.zip › life-3296331-supplementary.pdf]

A

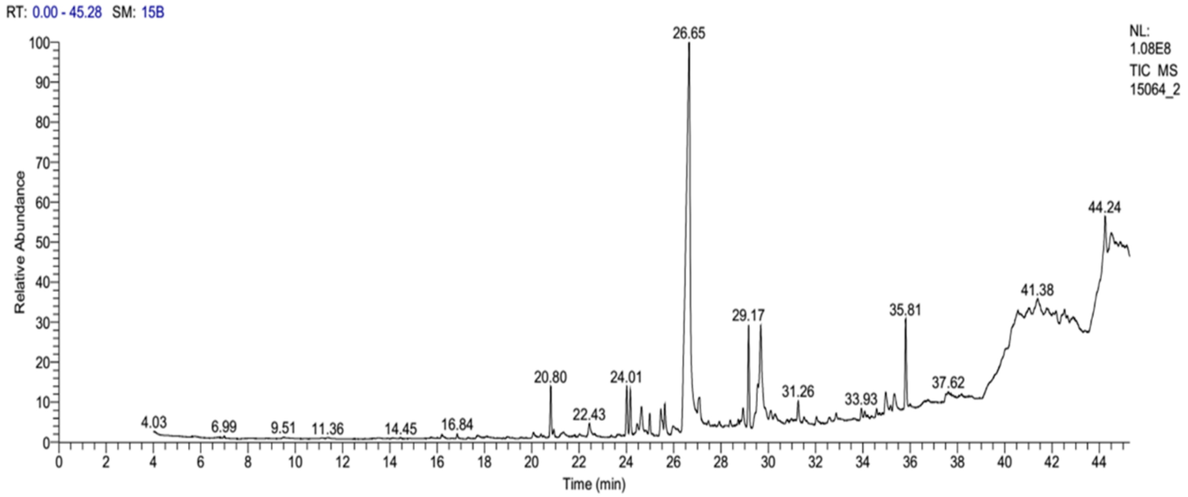

B 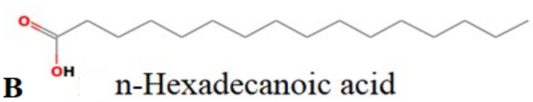

n-Hexadecanoic acid

C 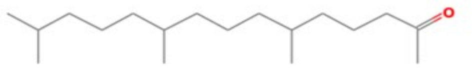

2-Pentadecanone, 6,10,14-trimethyl-

D 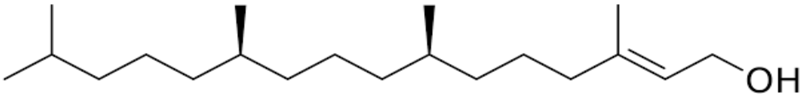

Phytol

E 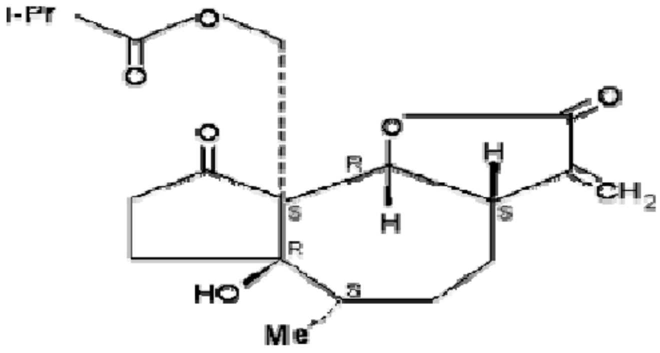

Isochiapin B

Fig S1. GC/MS chromatogram; **A)** *U. lactuca* (Methylene chloride extract), and structure, formula, molecular weight, CAS number and the relative abundance of the most four abundant metabolites **B**) n-Hexadecanoic acid **C**) 2-Pentadecanone, 6,10,14-trimethyl- **D**) Phytol E) Isochiapin B.

A

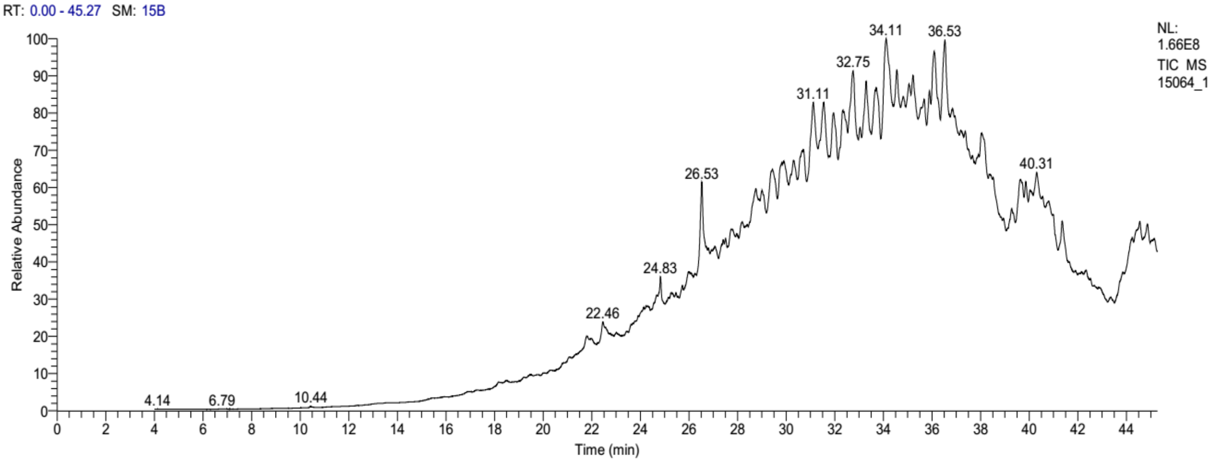

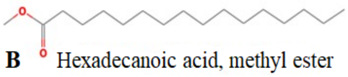

B Hexadecanoic acid, methyl ester

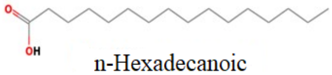

C n-Hexadecanoic

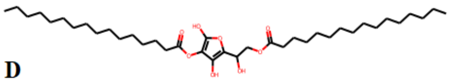

D l-(+)-Ascorbic acid 2,6-dihexadecanoate

E

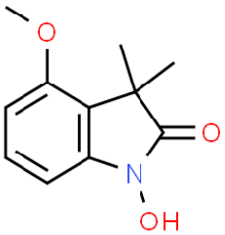

Indole-2-one,2,3-dihydro-N-hydroxy-4-methoxy-3,3-dimethyl-

F

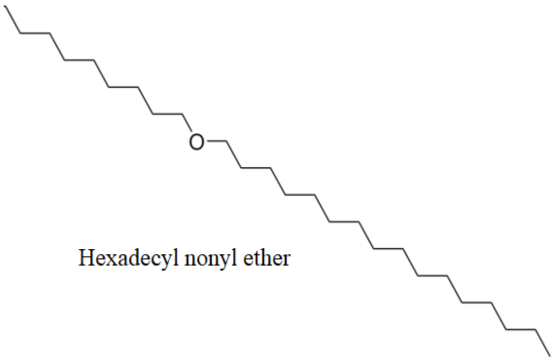

Hexadecyl nonyl ether

Fig S2. **A**) GC/MS chromatogram of acetone extract of *T. ornata* and the formula, molecular weight, CAS number and the relative abundance of the most abundant metabolites **B**) Hexadecanoic acid, methyl ester **C**) n-Hexadecanoic acid **D**) L-(+)-Ascorbic acid 2,6-dihexadecanoate and **E**) Indole-2-one,2,3-dihydro-N-hydroxy-4-methoxy-3,3-dimethyl- **F**) Hexadecyl nonyl ether
